# Supplementary material for: Barriers to the Utilization of Low-Vision Rehabilitation Services among Over-50-Year-Old People in East and Southeast Asian Regions: A Scoping Review
Source: Int J Environ Res Public Health. 2023 Dec 4;20(23):7141. doi: 10.3390/ijerph20237141 (PMC10706038; doi:10.3390/ijerph20237141)
Supplement: Supplementary file 1 [file ijerph-20-07141-s001.zip › 2.Appendix A_search strategy_revised.pdf]

Appendix A.

Table S1. Search strategy

Web of Science (Core collection)

Search date: 18. April. 2023

#	Searches	Results
1	TS=("eastren asia") OR TS=("east asia") OR TS=(china*) OR TS=(chinese*) OR TS=("hong kong") OR TS=(macau) OR TS=(tibet*) OR TS=(tibetan*) OR TS=(korea*) OR TS=(korean*) OR TS=(mongolia*) OR TS=(mongolian*) OR TS=(taiwan*) OR TS=(taiwanese*) OR TS=(japan*) OR TS=(japanese*) OR TS=("southeastern asia") OR TS=("southeast asia") OR TS=(borneo) OR TS=(bornean*) OR TS=(brunei*) OR TS=(bruneian*) OR TS=(cambodia*) OR TS=(cambodian*) OR TS=(indonesia*) OR TS=(indonesian*) OR TS=(laos*) OR TS=(laotian*) OR TS=(malaysia*) OR TS=(myanmar*) OR TS=(burmese*) OR TS=(philippines*) OR TS=(filipino*) OR TS=(singapore*) OR TS=(singaporean*) OR TS=(thailand*) OR TS=(thai*) OR TS=("timor leste*") OR TS=("east timorese*") OR TS=(vietnam*) OR TS=(vietnamese*)	2676564
2	TS=("visually impaired person*") OR TS=(blind) OR TS=(blindness) OR TS=("visually impaired") OR TS=("impaired vision") OR TS=("vision impairment") OR TS=("low vision") OR TS=("visual impairment") OR TS=("partially sighted") OR TS=("sight loss") OR TS=("eye problem*") OR TS=(disabilit*)	932698
3	TS=("self help device*") OR TS=(Smartphone) OR TS=("Health Services for Persons with Disabilities") OR TS=(rehabilitation) OR TS=("service animal*") OR TS=("sensory aid*") OR TS=("occupational therapy") OR TS=("activities of daily living") OR TS=("orientation and mobility") OR TS=("orientation and navigation") OR TS=("low vision care") OR TS=("vision rehabilitation") OR TS=("guide dog*") OR TS=("long term care") OR TS=(training) OR TS=(Braille) OR TS=("service dog*") OR TS=("white cane") OR TS=("supportive service") OR TS=(aid*) OR TS=("eye service") OR TS=("eye care") OR TS=("vision service") OR TS=("low vision	3123703

	rehabilitation") OR TS=("vision assistive") OR TS=(spectacle*) OR TS=(eyeglass*) OR TS=(glasses) OR TS=("magnifying glass") OR TS=("Smart-phone") OR TS=(ipad) OR TS=("electronic vision enhancement system*") OR TS=("closed circuit television magnif*") OR TS=("Large print") OR TS=("Writing guide") OR TS=(Typoscope) OR TS=("reading guide") OR TS=(Magnifier) OR TS=(Optician) OR TS=(Optometrist) OR TS=("Certified orthoptist") OR TS=("Smart sight") OR TS=("Digital Divide") OR TS=("Assistive Technolog*") OR TS=("Assistive Device*") OR TS=("Assistance Animal*") OR TS=("Seeing Eye Dog*") OR TS=("Alert Dog*") OR TS=("Assistance Dog*")	
4	TS=("facilities and services utilization") OR TS=("health services accessibility") OR TS=("Referral and Consultation") OR TS=("service use") OR TS=("service utilization") OR TS=("service uptake") OR TS=(barrier*) OR TS=(facilitator*) OR TS=(enabler*) OR TS=(access) OR TS=(accessibility) OR TS=("service access") OR TS=("service coverage") OR TS=(Referral) OR TS=(underutilization) OR TS=(underutilized) OR TS=(underuse) OR TS=(underused) OR TS=(availability) OR TS=(adherence) OR TS=(unavailability) OR TS=(inaccessibility) OR TS=(unmet) OR TS=("service provision") OR TS=("service delivery") OR TS=("Facilities Utilization") OR TS=("service Utilization") OR TS=(Consultation) OR TS=(gatekeeper*)	3088299
5	(LA=(Japanese)) OR JA=(English)	80380579
6	DOP=(2000-01-01/2023-03-31)	55341888
7	#1 AND #2 AND #3 AND #4 AND #5 AND #6	982
8	#1 AND #2 AND #3 AND #4 AND #5 AND #6 and Article (原著論文) （ドキュメントタイプ）	849

EBSCO (academic search ultimate)

Search date: 18. April. 2023

#	Searches	Search options	Results
1	AB"eastren asia" OR AB "east asia" OR AB china* OR AB chinese* OR AB "hong kong" OR AB macau OR AB tibet* OR AB tibetan* OR AB korea* OR AB korean* OR AB mongolia* OR AB mongolian* OR AB taiwan* OR AB taiwanese* OR AB japan* OR AB japanese* OR AB "southeastern asia" OR AB "southeast asia" OR AB borneo OR AB bornean* OR AB brunei* OR AB bruneian* OR AB cambodia* OR AB cambodian* OR AB indonesia* OR AB indonesian* OR AB laos* OR AB laotian* OR AB malaysia* OR AB myanmar* OR AB burmese* OR AB philippines* OR AB filipino* OR AB singapore* OR AB singaporean* OR AB thailand* OR AB thai* OR AB "timor leste*" OR AB "east timorese*" OR AB vietnam* OR AB vietnamese*	"拡張-同等のサブジェクトを適用	1410303
2	AB "visually impaired person*" OR AB blind OR AB blindness OR AB "visually impaired" OR AB "impaired vision" OR AB "vision impairment" OR AB "low vision" OR AB "visual impairment" OR AB "partially sighted" OR AB "sight loss" OR AB "eye problem*" OR AB disabilit* OR ((DE "PEOPLE with visual disabilities" OR DE "ACCESSIBLE design for people with visual disabilities" OR DE "BLIND people" OR DE "COMPUTERS & people with visual disabilities" OR DE "MAPS for people with visual disabilities" OR DE "OLDER people with visual disabilities" OR DE "MOBILITY of people with visual disabilities" OR DE "VISION disorders") OR (DE "PEOPLE with perceptual disabilities")) OR (DE "VISION disorders" OR DE "AMBLYOPIA" OR DE "BINOCULAR vision disorders" OR DE "BLINDNESS" OR DE "CHARLES Bonnet syndrome" OR DE "COLOR blindness" OR DE "COMPUTER vision syndrome" OR DE "HYPERMETROPIA" OR DE "LOW vision" OR DE "MEARES-lrlen syndrome" OR DE "MIGRAINE aura" OR DE "MONOCULAR vision" OR DE "REFRACTIVE errors" OR DE	同上	372264

	"SCOTOMA" OR DE "VISION disorders -- Social aspects" OR DE "EXOTROPIA" OR DE "EYE movement disorders")		
3	AB "self help device*" OR AB Smartphone OR AB "Health Services for Persons with Disabilities" OR AB rehabilitation OR AB "service animal*" OR AB "sensory aid*" OR AB "occupational therapy" OR AB "activities of daily living" OR AB "orientation and mobility" OR AB "orientation and navigation" OR AB "low vision care" OR AB "vision rehabilitation" OR AB "guide dog*" OR AB "long term care" OR AB training OR AB Braille OR AB "service dog*" OR AB "white cane" OR AB "supportive service" OR AB aid* OR AB "eye service" OR AB "eye care" OR AB "vision service" OR AB "low vision rehabilitation" OR AB "vision assistive" OR AB spectacle* OR AB eyeglass* OR AB glasses OR AB "magnifying glass" OR AB "Smart-phone" OR AB ipad OR AB "electronic vision enhancement system*" OR AB "closed circuit television magnif*" OR AB "Large print" OR AB "Writing guide" OR AB Typoscope OR AB "reading guide" OR AB Magnifier OR AB Optician OR AB Optometrist OR AB "Certified orthoptist" OR AB "Smart sight" OR AB "Digital Divide" OR AB "Assistive Technolog*" OR AB "Assistive Device*" OR AB "Assistance Animal*" OR AB "Seeing Eye Dog*" OR AB "Alert Dog*" OR AB "Assistance Dog*" OR (((DE "BRAILLE" OR DE "PRINTING for blind people" OR DE "BLIND people" OR DE "BLINDNESS" OR DE "BRAILLE books") OR (DE "LARGE type books" OR DE "BIG books")) OR (DE "LARGE type books")) OR (DE "SERVICES for blind people" OR DE "EMPLOYMENT of blind people" OR DE "INSTITUTIONAL care of blind people" OR DE "REHABILITAJION of blind people" OR DE "BLIND people")	同上	1352109
4	AB "facilities and services utilization" OR AB "health services accessibility" OR AB "Referral and Consultation" OR AB "service use" OR AB "service utilization" OR AB "service uptake" OR AB barrier* OR AB facilitator* OR AB enabler* OR AB access OR AB accessibility OR AB "service	同上	1407961

	access" OR AB "service coverage" OR AB Referral OR AB underutilization OR AB underutilized OR AB underuse OR AB underused OR AB availability OR AB adherence　OR AB unavailability OR AB inaccessibility OR AB unmet OR AB "service provision" OR AB "service delivery" OR AB "Facilities Utilization" OR AB "service Utilization"　OR AB Consultation OR AB gatekeeper*　OR ((DE "UTILIZATION　of services for older people")　OR (DE "UTILIZATION of hospital rehabilitation services" OR DE "HOSPITAL utilization")) OR (DE "HEALTH services accessibility" OR DE "ACCESS to primary care" OR DE "MEDICAL care wait times" OR DE "UNIVERSAL healthcare" OR DE "HEALTH programs")		
5	S1 AND S2 AND S3 AND S4	同上	278
6	S1 AND S2 AND S3 AND S4	同上＋限定-学術誌(査読); 出版日:20000101-20230331; 出版物タイプ:Academic Journal; 文献タイプ:Article; 言語:English, Japanese	220

MEDLINE (PubMed)

Search date: 19. April. 2023

#	Searches	Results
1	"asia, Eastern"[MeSH Terms] OR "china*"[All Fields] OR "chinese*"[All Fields] OR "hong kong"[All Fields] OR "macau"[All Fields] OR "tibet*"[All Fields] OR "tibetan*"[All Fields] OR "korea*"[All Fields] OR "korean*"[All Fields] OR "mongolia*"[All Fields] OR "mongolian*"[All Fields] OR "taiwan*"[All Fields] OR "taiwanese*"[All Fields] OR "japan*"[All Fields] OR "japanese*"[All Fields] OR "asia, southeastern"[MeSH Terms] OR "borneo*"[All Fields] OR "bornean*"[All Fields] OR "brunei*"[All Fields] OR "bruneian*"[All Fields] OR "cambodia*"[All Fields] OR "cambodian*"[All Fields] OR "indonesia*"[All Fields] OR "indonesian*"[All Fields] OR "laos*"[All Fields] OR "laotian*"[All Fields] OR "malaysia*"[All Fields] OR "malaysian*"[All Fields] OR "myanmar*"[All Fields] OR "burmese*"[All Fields] OR "philippines*"[All Fields] OR "filipino*"[All Fields] OR "singapore*"[All Fields] OR "singaporean*"[All Fields] OR "thailand*"[All Fields] OR "thai*"[All Fields] OR "timor leste*"[All Fields] OR "east timorese*"[All Fields] OR "vietnam*"[All Fields] OR "vietnamese*"[All Fields]	5,844,121
2	"visually impaired persons"[MeSH Terms] OR "blind"[All Fields] OR "blindness"[All Fields]OR "visually impaired"[All Fields] OR "impaired vision"[All Fields] OR "vision impairment"[All Fields] OR "low vision"[All Fields] OR "visual impairment"[All Fields] OR "partially sighted"[All Fields] OR "sight loss"[All Fields] OR "eye problem"[All Fields] OR "eye problems"[All Fields] OR "disabilit*"[All Fields]	620,997
3	"self help devices"[MeSH Terms] OR "Smartphone"[MeSH Terms] OR "Health Services for Persons with Disabilities"[MeSH Terms] OR "rehabilitation"[MeSH Terms] OR "service animals"[MeSH Terms] OR "sensory aids"[MeSH Terms] OR "rehabilitation"[Title/Abstract] OR "occupational therapy"[Title/Abstract] OR "activities of daily living"[Title/Abstract] OR "orientation and mobility"[Title/Abstract] OR "orientation and navigation"[Title/Abstract] OR "low vision care"[Title/Abstract] OR "vision rehabilitation"[Title/Abstract] OR "guide dogs"[Title/Abstract] OR "long term care"[Title/Abstract] OR "training"[Title/Abstract] OR "Braille"[Title/Abstract]	1,419,293

	OR "service dogs"[Title/Abstract] OR "white cane"[Title/Abstract] OR "supportive service"[Title/Abstract] OR "aid"[Title/Abstract] OR "aids"[Title/Abstract] OR "eye service"[Title/Abstract] OR "eye care"[Title/Abstract] OR "vision service"[Title/Abstract] OR "low vision rehabilitation"[Title/Abstract] OR "vision assistive "[Title/Abstract] OR "spectacle*"[Title/Abstract] OR "eyeglass*"[Title/Abstract] OR "glasses"[Title/Abstract] OR "magnifying glass"[Title/Abstract] OR "Smartphone"[Title/Abstract] OR "Smart-phone"[Title/Abstract] OR "ipad"[Title/Abstract] OR "electronic vision enhancement system*"[Title/Abstract] OR "closed circuit television magnif*"[Title/Abstract] OR "Large print"[Title/Abstract] OR "Writing guide"[Title/Abstract] OR "reading guide"[Title/Abstract] OR "Typoscope"[Title/Abstract] OR "Magnifier"[Title/Abstract] OR "Optometrist"[Title/Abstract] OR "Optician"[Title/Abstract] OR "Certified orthoptist"[Title/Abstract] OR "Smart sight"[Title/Abstract] OR "Digital Divide"[MeSH Terms]	
4	"facilities and services utilization"[MeSH Terms] OR "health services accessibility"[MeSH Terms] OR "Referral and Consultation"[MeSH Terms] OR "service use"[Title/Abstract] OR "service utilization"[Title/Abstract] OR "service uptake"[Title/Abstract] OR "barrier*"[Title/Abstract] OR "facilitator*"[Title/Abstract] OR "enabler*"[Title/Abstract] OR "access"[Title/Abstract] OR "accessibility"[Title/Abstract] OR "service access"[Title/Abstract] OR "service coverage"[Title/Abstract] OR "Referral"[Title/Abstract] OR "underutilization"[Title/Abstract] OR "underutilized"[Title/Abstract] OR "underuse"[Title/Abstract] OR "underused"[Title/Abstract] OR "availability"[Title/Abstract] OR " adherence"[Title/Abstract] OR "unavailability"[Title/Abstract] OR "inaccessibility"[Title/Abstract] OR "unmet"[Title/Abstract] OR " service provision"[Title/Abstract] OR "service delivery" [Title/Abstract]	1,524,981
5	"english"[Language] OR "japanese"[Language]	31,191,346
6	("2000/1/1"[Date - Publication] : "2023/3/31"[Date - Publication])	21,968,118
7	"editorial"[Publication Type] OR "congress"[Publication Type] OR "case reports"[Publication Type]	3,053,248
8	#1 AND #2 AND #3 AND #4 AND #5 AND #6	846

9	#8 NOT #7	828

Ichushi-Web (Japanese medical literature database)

Search date: 19. April. 2023

#	Searches	Results
1	(((東アジア/TH) and (DT=2000:2023 and (PT=症例報告・事例除く) and (PT=原著論文))) or ((東南アジア/TH) and (DT=2000:2023 and (PT=症例報告・事例除く) and (PT=原著論文))) or (((中国/TH or 中国/AL)) and (DT=2000:2023 and LA=日本語,英語 and (PT=症例報告・事例除く) and (PT=原著論文))) or (((香港/TH or 香港/AL)) and (DT=2000:2023 and LA=日本語,英語 and (PT=症例報告・事例除く) and (PT=原著論文))) or (((香港/TH or ホンコン/AL)) and (DT=2000:2023 and LA=日本語,英語 and (PT=症例報告・事例除く) and (PT=原著論文))) or (((マカオ/TH or マカオ/AL)) and (DT=2000:2023 and LA=日本語,英語 and (PT=症例報告・事例除く) and (PT=原著論文))) or (((チベット自治区/TH or チベット/AL)) and (DT=2000:2023 and LA=日本語,英語 and (PT=症例報告・事例除く) and (PT=原著論文))) or (((韓国/TH or 韓国/AL)) and (DT=2000:2023 and LA=日本語,英語 and (PT=症例報告・事例除く) and (PT=原著論文))) or (((モンゴル/TH or モンゴル/AL)) and (DT=2000:2023 and LA=日本語,英語 and (PT=症例報告・事例除く) and (PT=原著論文))) or (((台湾/TH or 台湾/AL)) and (DT=2000:2023 and LA=日本語,英語 and (PT=症例報告・事例除く) and (PT=原著論文))) or (((日本/TH or 日本/AL)) and (DT=2000:2023 and LA=日本語,英語 and (PT=症例報告・事例除く) and (PT=原著論文))) or (((ボルネオ島/TH or ボルネオ/AL)) and (DT=2000:2023 and LA=日本語,英語 and (PT=症例報告・事例除く) and (PT=原著論文))) or (((ブルネイ/TH or ブルネイ/AL)) and (DT=2000:2023 and LA=日本語,英語 and (PT=症例報告・事例除く) and (PT=原著論文))) or (((カンボジア/TH or カンボジア/AL)) and (DT=2000:2023 and LA=日本語,英語 and (PT=症例報告・事例除く) and (PT=原著論文))) or (((インドネシア/TH or インドネシア/AL)) and (DT=2000:2023 and LA=日本語,英語 and (PT=症例報告・事例除く) and (PT=原著論文))) or (((ラオス/TH or ラオス/AL)) and (DT=2000:2023 and LA=日本語,英語 and (PT=症例報告・事例除く) and (PT=原著論文))) or (((マレーシア/TH or マレーシア/AL)) and (DT=2000:2023 and LA=日本語,英語 and (PT=症例報告・事例除く) and (PT=原著論文))) or (((ミャンマー/TH or ミャンマー/AL)) and (DT=2000:2023 and LA=日本語,英語 and (PT=症例報告・事例除く) and (PT=原著論文))) or (((フィリピン/TH or フィリピン/AL)) and (DT=2000:2023 and LA=日本語,英語	439,446

	and (PT=症例報告・事例除く) and (PT=原著論文))) or (((シンガポール/TH or シンガポール/AL)) and (DT=2000:2023 and LA=日本語,英語 and (PT=症例報告・事例除く) and (PT=原著論文))) or (((タイ国/TH or タイ国/AL)) and (DT=2000:2023 and LA=日本語,英語 and (PT=症例報告・事例除く) and (PT=原著論文))) or (((東ティモール/TH or 東ティモール/AL)) and (DT=2000:2023 and LA=日本語,英語 and (PT=症例報告・事例除く) and (PT=原著論文))) or (((ベトナム/TH or ベトナム/AL)) and (DT=2000:2023 and LA=日本語,英語 and (PT=症例報告・事例除く) and (PT=原著論文))))	
2	(((視覚障害者/TH) and (DT=2000:2023 and LA=日本語,英語 and (PT=症例報告・事例除く) and (PT=原著論文))) or ((失明/TH) and (DT=2000:2023 and LA=日本語,英語 and (PT=症例報告・事例除く) and (PT=原著論文))) or ((低視力/TH) and (DT=2000:2023 and LA=日本語,英語 and (PT=症例報告・事例除く) and (PT=原著論文))) or ((視覚障害/TH) and (DT=2000:2023 and LA=日本語,英語 and (PT=症例報告・事例除く) and (PT=原著論文))) or ((障害/TA) and (DT=2000:2023 and LA=日本語,英語 and (PT=症例報告・事例除く) and (PT=原著論文))) or ((眼疾患/TH) and (DT=2000:2023 and LA=日本語,英語 and (PT=症例報告・事例除く) and (PT=原著論文))))	87,763
3	(((自助具/TH) and (DT=2000:2023 and LA=日本語,英語 and (PT=症例報告・事例除く) and (PT=原著論文))) or ((モバイルアプリケーション/TH) and (DT=2000:2023 and LA=日本語,英語 and (PT=症例報告・事例除く) and (PT=原著論文))) or ((スマートフォン/TH) and (DT=2000:2023 and LA=日本語,英語 and (PT=症例報告・事例除く) and (PT=原著論文))) or ((障害者保健医療サービス/TH) and (DT=2000:2023 and LA=日本語,英語 and (PT=症例報告・事例除く) and (PT=原著論文))) or ((リハビリテーション/TH) and (DT=2000:2023 and LA=日本語,英語 and (PT=症例報告・事例除く) and (PT=原著論文))) or ((身体障害者補助動物/TH) and (DT=2000:2023 and LA=日本語,英語 and (PT=症例報告・事例除く) and (PT=原著論文))) or ((感覚補助具/TH) and (DT=2000:2023 and LA=日本語,英語 and (PT=症例報告・事例除く) and (PT=原著論文))) or ((盲導犬/TH) and (DT=2000:2023 and LA=日本語,英語 and (PT=症例報告・事例除く) and (PT=原著論文))) or ((点字/TH) and (DT=2000:2023 and LA=日本語,英語 and (PT=症例報告・事例除く) and (PT=原著論文))) or ((眼鏡/TH) and (DT=2000:2023 and LA=日本語,英語 and (PT=症例報告・事例除く) and (PT=原著論文))) or ((デジタルデバイド/TH) and (DT=2000:2023 and LA=日本語,英語 and (PT=症例報告・事例除く) and (PT=原著論文)))	64,588

	or ((リハビリテーション/TA) and (DT=2000:2023 and LA=日本語,英語 and (PT=症例報告・事例除く) and (PT=原著論文))) or ((作業療法/TA) and (DT=2000:2023 and LA=日本語,英語 and (PT=症例報告・事例除く) and (PT=原著論文))) or ((日常生活活動/TA) and (DT=2000:2023 and LA=日本語,英語 and (PT=症例報告・事例除く) and (PT=原著論文))) or ((歩行訓練士/TA) and (DT=2000:2023 and LA=日本語,英語 and (PT=症例報告・事例除く) and (PT=原著論文))) or ((ロービジョンケア/TA) and (DT=2000:2023 and LA=日本語,英語 and (PT=症例報告・事例除く) and (PT=原著論文))) or ((ビジョンリハビリテーション/TA) and (DT=2000:2023 and LA=日本語,英語 and (PT=症例報告・事例除く) and (PT=原著論文))) or ((視覚リハビリテーション/TA) and (DT=2000:2023 and LA=日本語,英語 and (PT=症例報告・事例除く) and (PT=原著論文))) or ((トレーニング/TA) and (DT=2000:2023 and LA=日本語,英語 and (PT=症例報告・事例除く) and (PT=原著論文))) or ((介助犬/TA) and (DT=2000:2023 and LA=日本語,英語 and (PT=症例報告・事例除く) and (PT=原著論文))) or ((白杖/TA) and (DT=2000:2023 and LA=日本語,英語 and (PT=症例報告・事例除く) and (PT=原著論文))) or ((アイケア/TA) and (DT=2000:2023 and LA=日本語,英語 and (PT=症例報告・事例除く) and (PT=原著論文))) or ((ビジョンケア/TA) and (DT=2000:2023 and LA=日本語,英語 and (PT=症例報告・事例除く) and (PT=原著論文))) or ((ロービジョン/TA) and (DT=2000:2023 and LA=日本語,英語 and (PT=症例報告・事例除く) and (PT=原著論文))) or ((遮光眼鏡/TA) and (DT=2000:2023 and LA=日本語,英語 and (PT=症例報告・事例除く) and (PT=原著論文))) or ((老眼鏡/TA) and (DT=2000:2023 and LA=日本語,英語 and (PT=症例報告・事例除く) and (PT=原著論文))) or ((拡大鏡/TA) and (DT=2000:2023 and LA=日本語,英語 and (PT=症例報告・事例除く) and (PT=原著論文))) or ((スマートフォン/TA) and (DT=2000:2023 and LA=日本語,英語 and (PT=症例報告・事例除く) and (PT=原著論文))) or ((ipad/TA) and (DT=2000:2023 and LA=日本語,英語 and (PT=症例報告・事例除く) and (PT=原著論文))) or ((電子式視覚機能拡張システム/TA) and (DT=2000:2023 and LA=日本語,英語 and (PT=症例報告・事例除く) and (PT=原著論文))) or ((拡大読書器/TA) and (DT=2000:2023 and LA=日本語,英語 and (PT=症例報告・事例除く) and (PT=原著論文))) or ((タイポスコープ/TA) and (DT=2000:2023 and LA=日本語,英語 and (PT=症例報告・事例除く) and (PT=原著論文))) or ((眼鏡士/TA) and (DT=2000:2023 and LA=日本語,英語 and (PT=症例報告・事例除く) and (PT=原著論文))) or ((視能訓練士/TA) and (DT=2000:2023 and LA=日本語,英語 and (PT=症例報告・事例除く) and (PT=原著論文))) or ((スマートサイト/TA) and	

	(DT=2000:2023 and LA=日本語,英語 and (PT=症例報告・事例除く) and (PT=原著論文))) or ((補装具/TH) and (DT=2000:2023 and LA=日本語,英語 and (PT=症例報告・事例除く) and (PT=原著論文))) or ((介護サービス/TH) and (DT=2000:2023 and LA=日本語,英語 and (PT=症例報告・事例除く) and (PT=原著論文))))	
4	"(((施設とサービスの利用状況/TH) and (DT=2000:2023 and LA=日本語,英語 and (PT=症例報告・事例除く) and (PT=原著論文))) or ((保健医療サービス利用可能性/TH) and (DT=2000:2023 and LA=日本語,英語 and (PT=症例報告・事例除く) and (PT=原著論文))) or ((紹介と相談/TH) and (DT=2000:2023 and LA=日本語,英語 and (PT=症例報告・事例除く) and (PT=原著論文))) or ((サービス利用/TA) and (DT=2000:2023 and LA=日本語,英語 and (PT=症例報告・事例除く) and (PT=原著論文))) or ((障壁/TA) and (DT=2000:2023 and LA=日本語,英語 and (PT=症例報告・事例除く) and (PT=原著論文))) or ((阻害要因/TA) and (DT=2000:2023 and LA=日本語,英語 and (PT=症例報告・事例除く) and (PT=原著論文))) or ((阻害因子/TA) and (DT=2000:2023 and LA=日本語,英語 and (PT=症例報告・事例除く) and (PT=原著論文))) or ((促進要因/TA) and (DT=2000:2023 and LA=日本語,英語 and (PT=症例報告・事例除く) and (PT=原著論文))) or ((促進因子/TA) and (DT=2000:2023 and LA=日本語,英語 and (PT=症例報告・事例除く) and (PT=原著論文))) or ((アクセス/TA) and (DT=2000:2023 and LA=日本語,英語 and (PT=症例報告・事例除く) and (PT=原著論文))) or ((アクセシビリティ/TA) and (DT=2000:2023 and LA=日本語,英語 and (PT=症例報告・事例除く) and (PT=原著論文))) or ((サービスアクセス/TA) and (DT=2000:2023 and LA=日本語,英語 and (PT=症例報告・事例除く) and (PT=原著論文))) or ((サービス利用率/TA) and (DT=2000:2023 and LA=日本語,英語 and (PT=症例報告・事例除く) and (PT=原著論文))) or ((照会/TA) and (DT=2000:2023 and LA=日本語,英語 and (PT=症例報告・事例除く) and (PT=原著論文))) or ((低利用/TA) and (DT=2000:2023 and LA=日本語,英語 and (PT=症例報告・事例除く) and (PT=原著論文))) or ((利用可能性/TA) and (DT=2000:2023 and LA=日本語,英語 and (PT=症例報告・事例除く) and (PT=原著論文))))	12852
5	#1 AND #2 AND #3 AND #4	260
